# In a large Juvenile Idiopathic Arthritis (JIA) cohort, concomitant celiac disease is associated with family history of autoimmunity and a more severe JIA course: a retrospective study

**DOI:** 10.1186/s12969-022-00689-4

**Published:** 2022-04-22

**Authors:** Roberta Naddei, Simona Di Gennaro, Alfredo Guarino, Riccardo Troncone, Maria Alessio, Valentina Discepolo

**Affiliations:** 1 Department of Translational Medical Sciences, Section of Pediatrics, University of Naples Federico II, Via Sergio Pansini 5, 80131 Naples, Italy; 2 European Laboratory for the Investigation of Food Induced Diseases (ELFID), University of Naples Federico II, Naples, Italy

**Keywords:** Celiac disease, Juvenile idiopathic arthritis, Autoimmunity, Children, Anti-tissue transglutaminase antibodies, Screening

## Abstract

**Background:**

A higher prevalence of celiac disease (CD) has been reported in patients with juvenile idiopathic arthritis (JIA) compared to the general population. Factors related to the increased risk of co-occurrence and associated disease course have not been fully elucidated. Aims of this study were to determine the prevalence of CD in a large Southern Italian cohort of children with JIA, describe their clinical features and disease course and investigate risk factors associated with their co-occurrence.

**Findings:**

Demographic, clinical and laboratory data of all patients with JIA admitted to our Pediatric Rheumatology Unit from January 2001 to June 2019, who underwent CD screening, were retrospectively extracted from clinical charts and analyzed. Eight of 329 JIA patients were diagnosed with CD, resulting in a prevalence higher than the general Italian population (2.4% vs 0.93%, *p* < 0.05). Familiarity for autoimmunity was reported by 87.5% patients with JIA and CD compared to 45.8% of those without CD (*p* < 0.05). 87.5% patients with JIA and CD required both a conventional Disease Modifying Anti-Rheumatic Drug (DMARD) and a biological DMARD over time compared to 36.4% of those without CD (*p* < 0.05).

**Conclusion:**

A higher CD prevalence was found in a large JIA cohort, supporting the need for CD screening in all JIA children, especially those with a family history of autoimmunity, found to be associated with the co-occurrence of the two diseases. This is clinically relevant since patients with CD and JIA more often required a step-up therapy, suggesting a more severe JIA clinical course.

**Supplementary Information:**

The online version contains supplementary material available at 10.1186/s12969-022-00689-4.

## Background

Autoimmune disorders often share immune pathogenic mechanisms and predisposing factors, including common genetic susceptibility and environmental triggers [1]. Celiac disease (CD) is an autoimmune disorder elicited by dietary gluten, characterized by high titers of anti-tissue transglutaminase2 (anti-tTG) antibodies and the development of a small intestinal enteropathy that revert on a gluten-free diet (GFD). The prevalence of CD has been reported to be consistently higher in patients with juvenile idiopathic arthritis (JIA) in comparison to the general population, however not negligible variations have been observed in distinct geographic locations [2–11].

Several reports investigated the occurrence of CD in JIA patients, reporting a prevalence ranging from 0.7% in a Finnish cohort [5] to 6.6% in Florence, Italy [3]. Four Italian studies, all conducted in Northern centers, indicate a prevalence of 2.8% or higher [2–4, 8]. The lack of data on the risk factors associated with CD and JIA overlap prompted us to investigate the co-occurrence of these two diseases in our JIA pediatric cohort looking for potential predisposing factors. This represents the largest study looking at CD prevalence and predisposing factors in JIA patients in Italy, and the first in the Southern area of the country. Understanding the extent of the co-occurrence and identifying factors associated with an increased susceptibility could be relevant to implement case-finding strategies in specific at-risk populations.

## Methods

A single-center retrospective study was conducted by reviewing the medical records of all patients diagnosed with JIA according to the International League of Associations for Rheumatology (ILAR) criteria [12], admitted to the Pediatric Rheumatology Unit of the University of Naples Federico II from January 2001 to June 2019. All 329 patients underwent serological screening for CD, independently from the presence of suggestive symptoms. Patients whose clinical chart could not be retrieved were excluded. The study protocol was approved by the University of Naples Federico II Ethical Committee. For each patient, age at diagnosis, JIA subtype, family history for autoimmune diseases in first and second-degree relatives, co-occurrence of other autoimmune disorders, medications and laboratory data (Table 1) were extracted from charts and inserted into a secreted database. JIA patients were usually evaluated every three-six months with physical examination including joint assessment and laboratory investigations. Assessment of disease activity was based on the active joint count (swollen or both tender and restricted joints), the presence of morning stiffness and inflammatory markers values. Therapy was adjusted or changed according to disease activity fluctuations and/or drug adverse events. CD screening was systematically carried out by measuring serum anti-tTG IgA antibodies at the time of JIA diagnosis and thereafter annually. Total IgA levels were assessed at diagnosis to exclude IgA deficiency. CD was diagnosed according to the European Society of Pediatric Gastroenterology Hepatology and Nutrition (ESPGHAN) guidelines [13], combining clinical, serological, histological and genetic criteria. For CD patients, gastrointestinal and extra-intestinal symptoms, age at CD diagnosis, anti-tTG and anti-endomysium (EMA) antibodies levels, small intestinal pathology report, including Marsh score and human leukocyte antigen (HLA)-DQ2/DQ8 typing were also collected (Table 2).

**Table 1:** Clinical and demographic features of our cohort of children with JIA

Total patients with JIA		329
**Sex, N (%)**	F	246 (74.8)
	M	83 (25.2)
**Median Age, years (IQR)**	At JIA onset	4 (2.2–7.8)
	Present	12.5 (9.1–16.1)
**Follow-up duration, years (IQR)**		5.1 (2.7–8.9)
**JIA subtypes, N (%)**	Oligoarticular	215 (65.3)
	RF negative polyarticular	54 (16.4)
	RF positive polyarticular	3 (0.9)
	Systemic	29 (8.8)
	Psoriatic	8 (2.4)
	ERA	5 (1.5)
	Undifferentiated	15 (4.6)
**Family history of** **Autoimmunity, N (%)**	Yes	154 (46.8)
	No	175 (53.2)
**Co-occurrence of** **autoimmune disorders, N (%)**	Uveitis	48 (14.6)
	Presence of thyroid antibodies	23 (7)
	Autoimmune thyroiditis	20 (6.1)
	Celiac disease	8 (2.4)
	IBD	4 (1.2)
	Type 1 diabetes	1 (0.3)
**Therapy, N (%)**^**a**^	NSAID and/or intra-articular CS only	81 (24.6)
	MTX and/or other cDMARDs	240 (72.9)
	bDMARDs	134 (40.7)

*JIA* Juvenile Idiopathic Arthritis, *N* Number, *IQR* Interquartile Range, *ERA* Enthesitis-Related Arthritis, *IBD* Inflammatory Bowel Diseases, *IBD* Inflammatory Bowel Disease, *NSAID* Nonsteroidal Anti-inflammatory Drug, *CS* Corticosteroid, *MTX* Methotrexate, *cDMARDs* conventional Disease-Modifying Anti-Rheumatic Drugs, *bDMARDs* biological Disease-Modifying Anti-Rheumatic Drugs, *RF* rheumatoid factor

^a^Patients were classified according to JIA treatment administered during the overall disease course

**Table 2:** Clinical and laboratory features of patients with CD and JIA

	**Patient 1**	**Patient 2**	**Patient 3**	**Patient 4**	**Patient 5**	**Patient 6**	**Patient 7**	**Patient 8**
Sex	F	F	M	M	F	F	F	F
Current age (years)	16.5	16.6	13.8	8.4	14.1	21.6	13.5	12.1
JIA subtype	Polyarticular	Undifferentiated	Systemic	Oligoarticular	Oligoarticular	Undifferentiated	Polyarticular	Undifferentiated
Age of JIA onset (years)	4.3	4.1	5	6.5	4.5	3.4	13	3
ANA ( ±)	-	+	+	-	+	-	-	-
Follow-up duration (years)	12.2	12.5	8.8	1.9	9.6	15.2	0.5	9.1
Age at CD diagnosis (years)	5	14.6	10.9	6.10	5.2	3.6	9.7	3.9
Anti-tTG (fold increase over ULN)	125	10	2.3	11	7.5	1.8	7.6	3
EMA ( ±)	+	+	-	+	+	+	+	+
Small intestinal biopsy	+	n.d.	+	+	+	+	n.d.	+
Marsh classification	T3b\c	n.d.	T3c	T3c	T3c	T3b	n.d.	T0
HLA-DQ2\-DQ8	n.d.	DQ2.5	DQ2 and DQ8	n.d.	n.d.	n.d.	n.d.	DQ2
Gastrointestinal symptoms	No	No	Yes	No	No	No	Yes	Yes
Extra-intestinal symptoms of CD	No	No	Yes	No	No	No	Yes	No
JIA therapy at CD diagnosis	NSAID	ADA	PDN INDO; CsA; TCZ	NSAID	MTX	MTX	NA	NA^a^
Relapse upon GFD	Yes	Yes	No	Yes	Yes	Yes	NA	Yes
Positive family history of autoimmune disease	No	Yes	Yes	Yes	Yes	Yes	Yes	Yes
Family history of autoimmune disease specifics	NA	Psoriasis	Rheumatoid arthritis, type 1 diabetes	Thyroiditis	Thyroiditis, vitiligo	Psoriasis	Psoriasis, CD	Psoriasis
Co-occurrence of other autoimmune disorders	No	Uveitis	No	No	No	No	No	Uveitis

*NA* Not Applicable, *nd* not done, *CD* Celiac Disease, *JIA* Juvenile Idiopathic Arthritis, *ANA* Antinuclear Antibody, *Anti-tTG* Anti-transglutaminase antibodies IgA, *ULN* Upper Limit of Normal, *EMA* anti-Endomysial Antibodies, *HLA* Human Leukocyte Antigen, *GFD* Gluten Dree Diet, *NSAID* Non-Steroidal Anti-inflammatory Drugs, *ADA* Adalimumab, *PDN* Prednisone, *INDO* Indomethacin, *CsA* Cyclosporin, *TCZ* Tocilizumab, *MTX* Methotrexate,

^a^Patient 8 was off-therapy at CD diagnosis

Continuous variables were presented as median (interquartile range [IQR]) while categorical variables as percentage. The one-sample proportion test was used to compare CD prevalence in our cohort to the general population. Differences between JIA patients with or without CD were analyzed by Fisher’s exact test for categorical variables and Mann–Whitney U test for continuous ones. All statistical tests were two-sided and considered statistically significant with a *p*-value below 0.05. Data were analyzed using IMB SPSS Macintosh, Version 25.0 (Armonk, NY: IBM Corp).

## Results

Three hundred twenty-nine patients with JIA with a current median age 12.5 years (IQR 9.1–16.1) were included in our retrospective analysis: 246 females (74.8%) and 83 males (25.2%). Of those, 225 (68.4%) presented with an oligoarticular, 62 (18.9%) polyarticular, 30 (9.1%) systemic, 7 (2.1%) psoriatic subtype and 5 (1.5%) enthesitis-related arthritis as indicated in Table 1. Median age at JIA onset was 4 years (IQR: 2.2–7.8). 154 patients (46.8%) had a positive family history of autoimmunity, including rheumatological (rheumatoid arthritis, spondyloarthropathy, systemic lupus erythematosus, scleroderma) and non-rheumatological diseases (CD, type 1 diabetes, psoriasis, autoimmune thyroiditis, vitiligo and inflammatory bowel diseases).

Eight patients (2.4%) displayed positive CD-specific autoimmune markers (positive anti-tTG antibodies) falling into the CD spectrum. Of those, seven were diagnosed with full blown and one with Potential CD (Table 2). Of those, five fulfilled the ESPGHAN guidelines [13], while patient 2 and 7 were diagnosed based on positive CD-specific serology: anti-tTG IgA respectively 10 and 7 times above the upper limit of normal (ULN) and positive EMA, in the absence of histological assessment, since the families refused the upper gastro-intestinal endoscopy. Patient 8 was diagnosed with Potential CD based on positive serology (both anti-tTG and EMA) and normal intestinal architecture, however a GFD was prescribed for the presence of gastrointestinal symptoms. Gastrointestinal symptoms of patient 8, as well as those of patient 3 and 7, resolved upon GFD along with normalization of serology, further supporting their gluten-dependency. The diagnosis of patient 2 and 8 were further supported by the presence of the HLA-DQ2 haplotype.

None of CD patients presented also with type 1 diabetes nor with autoimmune thyroid disease.

The prevalence of CD in our JIA cohort (2.4%) was higher than that reported in Italy over the same period of time [14], ranging from 0.69 to 1.25% (ave. 0.93%, *p* < 0.05). Importantly, the result didn’t change when considering all CD patients (8/329, 2.4%) or only full-blown CD (7/329, 2.1%, *p* < 0.05).

There was no association between CD and age at JIA onset. Notably, 7 out of 8 patients (87.5%) with CD and JIA had at least one first or second-degree relative with an autoimmune disorder compared to 45.8% of those with JIA without CD (*p* < 0.05, Table 3), suggesting that a positive family history of autoimmunity can be a risk factor for concomitant CD. Since 3/8 patients affected by CD had a first-degree relative with psoriasis, undifferentiated arthritis resulted more common in children with CD compared to the others (37.5% vs 3.7%, *p* = 0.004, Table 3), while the oligoarticular subtype was more frequent in JIA patients without CD (66.4% vs 25%, *p* = 0.02, Table 3). Interestingly, we observed a higher prevalence of uveitis in patients with JIA and CD compared to those without CD, although not statistically significant (25% vs 14.3%, *p* = 0.33), probably due to the low number of patients with the three conditions (Table 3).

**Table 3:** Comparison of clinical features between JIA patients with and without CD

	**JIA patients without CD** ***N* = 321**	**JIA patients with CD** ***N* = 8**	***P*-value§**
Sex (female), n (%)	240 (74.8)	6 (75)	0.45
Age at JIA diagnosis, yrs, median (IQR)	4 (2.2–7.9)	4.4 (3.6–6.1)	0.32‡
JIA Subtype, n (%)			
Oligoarticular	213 (66.4)	2 (25)	0.02*
Polyarticular	55 (17.1)	2 (25)	0.63
Systemic	28 (8.7)	1 (12.5)	0.53
Undifferentiated	12 (3.7)	3 (37.5)	0.004*
Follow-up duration, yrs, median (IQR)	5 (2.7–8.8)	9.4 (3.6–12.4)	0.13‡
Uveitis, n (%)	46 (14.3)	2 (25)	0.33
Family history of autoimmunity, n (%)	147 (45.8)	7 (87.5)	0.03*
ANA positivity, n (%)	125 (38.9)	3 (37.5)	1
DMARD therapy, n (%)			
cDMARDs	233 (72.6)	7 (87.5)	0.7
bDMARDs	127 (39.6)	7 (87.5)	0.009*
cDMARDs + bDMARDs	117 (36.4)	7 (87.5)	0.005*

*JIA* Juvenile Idiopathic Arthritis, *CD* Celiac Disease, *IQR* Interquartile Range, *yrs* years, *ANA* Antinuclear Antibody, *DMARD* Disease-modifying Anti-rheumatic Drug, *cDMARDs* conventional Disease-modifying Anti-rheumatic Drugs, *bDMARDs* biological Disease-modifying Anti-rheumatic Drugs

^§^ By Fisher’s exact test unless otherwise specified. ‡ By Mann–Whitney U test. *Statistically significant

In one patient CD diagnosis preceded JIA onset, despite the ongoing strict GFD. The other seven patients developed JIA first and then CD, after a median time of 8.4 months (6.6–40.8). Most of those (5/7, 72%) were asymptomatic and CD was diagnosed following the routine serological screening that all JIA patients undergo in our clinic.

Out of 329 JIA patients, 24.6% were treated with intra-articular corticosteroids or nonsteroidal anti-inflammatory (NSAID), while the remaining 75.4% with disease-modifying anti-rheumatic drugs (DMARDs) (Table 1). Concerning the pharmacological treatment for JIA up to CD diagnosis there was no difference when comparing patients with or without CD (Table S1). As expected, in the majority of patients (6/9, 66.7%), CD developed regardless the ongoing therapies for JIA, including NSAID drugs, conventional or biological disease-modifying anti-rheumatic drugs (cDMARDs, bDMARDs). None of the patients with CD and JIA was able to discontinue the ongoing therapy after starting the GFD. In addition, despite the GFD, JIA relapsed in six patients and five of them required to start a new DMARD, suggesting that GFD did not ameliorate the disease course. Notably, 7/8 patients with CD and JIA required both a cDMARD and a bDMARD during follow-up compared to 117/321 of those without CD (87.5% vs 36.4%, *p* = 0.005, Table 3), suggesting a more severe JIA course in subjects with both conditions.

## Discussion

The present study supports and extends previous findings from smaller cohorts documenting the higher prevalence of CD in children and adolescents with JIA. Moreover, it indicates that CD and JIA co-occurrence is more frequent in patients with a positive family history of autoimmune diseases and often associated with a more severe JIA course.

Together with the one described by Pohjankoski and colleagues including 417 Finnish patients [5], our cohort is the largest so far investigated for JIA and CD co-occurrence. In contrast to their study, reporting a 0,7% CD prevalence among JIA patients, but in line with other reports [2–4, 8, 9], we found a prevalence of 2,4%, more than twice higher than that of CD in the general population in Italy [14], as well as in Europe [15]. Similarly, Oman and colleagues found a CD prevalence of 2,8% among 216 Swedish JIA patients [10]. In contrast to what observed in northern European countries [5, 10], a small Brazilian study found 4 in 45 (8,8%) JIA patients to have positive CD-specific serology, however no biopsy was performed [16]. In contrast, no CD case was found in two small JIA cohorts from the Middle East [7, 11].

Despite some limitations, with our 329 JIA patients, we describe here the second largest pediatric JIA cohort ever investigated for CD prevalence and the largest in Italy. In line with our findings, referring to a Southern Italian population, previous studies conducted in Northern Italy consistently reported an increased rate of CD among JIA patients. In particular, Lepore [2] found that 4/119 (3,3%) JIA patients had positive CD serology, of whom three presented also with small intestinal villous atrophy (1,6%), while Alpigiani reported a 2,8% prevalence among 108 JIA children [4]. A higher CD prevalence was reported by Stagi and colleagues (6,6%), albeit their screening included also IgA anti-gliadin antibodies [3], currently considered not specific for CD. The reported differences might be explained by geographical factors, inclusion criteria and screening tests performed.

In line with previous data [9, 10], at the time of CD diagnosis, most patients were asymptomatic, thus underlining the importance of systematic screening for CD among JIA patients even in the absence of suggestive symptoms.

In our cohort, all but one patient developed JIA before the onset of CD, that occurred most often during the first year after JIA onset. The close relationship in time between the two diagnosis may result from a combination of different factors including common genetic predisposition, an ongoing inflammatory response related to the first autoimmune disease (often requiring months to decline) and possibly common environmental triggers such as infections or dietary factors [17]. Some of those factors may also explain the strong familial aggregation.

When looking for factors associated with the co-occurrence of CD and JIA in our cohort, we found that a family history of autoimmunity was positively associated with their overlap. This was not related to one specific autoimmune condition, suggesting that predisposing factors shared across autoimmune disorders may contribute to both CD and JIA [1]. Our finding prompts to systematically screen JIA children, in particular those who have at least one first or second degree relative with an autoimmune disorder, and especially during the first year of JIA course.

Notably, we also observe an increased prevalence of autoimmune thyroid disease and type 1 diabetes in our JIA cohort compared to the general population, further supporting a role of shared genetic predisposing factors and environmental triggers across distinct autoimmune conditions.

Since only about 30% of JIA patients carry the HLA-DQ2 and DQ8 haplotypes, required for CD development [13], some authors suggested to perform HLA typing as a first-line screening in all JIA patients to identify those susceptible for CD prior to serological testing [9]. Whether this strategy may be more efficient than first-line serologic screening is debated [18].

Non-HLA genetic loci harboring genes related to the immune response have been associated to both JIA and CD susceptibility, including the 4q27 locus, harboring both the interleukin(*IL)2* and *IL21* genes [19, 20], two key cytokines involved in T cell function and autoimmunity [21, 22]. Variants of the Protein Tyrosine Phosphatase Non-Receptor Type 2 (*PTPN2)* gene, key regulator in immune cell signaling, have been associated with both diseases [23]. Finally, C–C Motif Chemokine Receptor 3 (*CCR3)* region (3p21), already linked to familiarity of autoimmunity among children with type I diabetes [24], has been recently associated with CD and JIA [19, 25]. Whether those or other gene variants are present in our patients with JIA and CD and their families is under investigation.

Untreated CD could lead to long-term increased morbidity and mortality, however, whether GFD can reduce the risk of developing other autoimmune diseases is debated [26]. In addition to JIA, joint involvement has been described in 5–10% CD patients, including subclinical synovitis or non-erosive arthritis in children, and arthralgia in adolescents [27]. The pathogenesis of these rheumatological manifestations in CD and their response to the GFD remains elusive. However, a lower prevalence of joint effusion [28] and symptoms [2, 4] has been reported in treated compared to untreated CD patients. Whether GFD downregulates pro-inflammatory responses leading to autoimmunity needs to be demonstrated. In contrast with this hypothesis, GFD did not improve the clinical course of our JIA patients, regardless of dietary compliance. On the contrary, to achieve clinical remission, our patients with JIA and CD required a step-up therapy with a bDMARDs more frequently than JIA patients without CD, suggesting that concomitant CD was associated with a more severe JIA course, independently of the activity of the intestinal disease. The reasons behind this observation require further exploration, we might only postulate that the ongoing inflammation caused by two concomitant autoimmune diseases, along with genetic predisposing factors that may render the joint inflammation more aggressive, might concur. Notably, immunomodulatory treatments for JIA had no role in preventing CD onset in our population, in fact JIA patients treated with either cDMARD or bDMARDs developed CD down the line.

### Limitations

Despite the large JIA cohort, the sample size of patients with both JIA and CD is small, thus limiting some analysis of their main characteristics. Some analysis might be also limited by the retrospective nature of the study. We do not report the disease activity for all patients, yet we indicate the general criteria used in our center. Lastly, we acknowledge that patients with JIA and CD have a longer follow-up than those with JIA alone, however, since most of CD diagnosis occurred within the first year after JIA onset, this should not represents a bias.

## Conclusions

In conclusion, we present the second largest pediatric cohort of patients with JIA investigated for co-occurrence with other autoimmune diseases, the largest in Italy, reporting a 2.58-fold increased prevalence of CD compared to the general population and showing that a family history of autoimmunity increases the risk of co-occurrence. These results underline the importance of CD screening in pediatric JIA patients. This is particularly relevant, since the clinical course of JIA appears to be more aggressive in patients with concomitant CD, who often require a step-up therapy. Whether those patients would benefit from an early introduction of a biologic drug needs to be explored. Future studies will test whether a first-line genetic testing followed by CD-specific serological screening would be more effective than a first-line serological screening.

## Supplementary Information

**Additional file 1: Table S1. **Pharmacological treatment for JIA in patients with and without CD. 

## Data Availability

All the clinical data are available with the authors upon request.

## Abbreviations

ANA: Antinuclear antibody
Anti-tTG: Anti-tissue transglutaminase2
CCR3: C–C motif chemokine Receptor 3
DMARD: Disease-Modifying Anti-Rheumatic drug
bDMARDs: Biological Disease-Modifying Anti-Rheumatic Drugs
cDMARDs: Conventional Disease-Modifying Anti-Rheumatic drugs
EMA: Anti-endomysium antibodies
ERA: Enthesitis-Related Arthritis
ESPGHAN: European Society of Pediatric Gastroenterology Hepatology and Nutrition
GFD: Gluten free diet
HLA: Human leukocyte antigen
IBD: Inflammatory Bowel Diseases
IL: Interleukin
ILAR: International League of Associations for Rheumatology
IQR: Interquartile range
JIA: Juvenile idiopathic arthritis
NSAID: Nonsteroidal anti-inflammatory drugs
PTPN2: Protein Tyrosine Phosphatase Non-Receptor Type 2
