# Supplementary material for: In a large Juvenile Idiopathic Arthritis (JIA) cohort, concomitant celiac disease is associated with family history of autoimmunity and a more severe JIA course: a retrospective study
Source: Pediatr Rheumatol Online J. 2022 Apr 22;20:31. doi: 10.1186/s12969-022-00689-4 (PMC9034531; doi:10.1186/s12969-022-00689-4)
Supplement: Supplementary file 1 — Additional file 1: Table S1. Pharmacological treatment for JIA in patients with and without CD. [file 12969_2022_689_MOESM1_ESM.docx]

**Table S1. Pharmacological treatment for JIA in patients with and without CD.**

| **JIA pharmacological treatments, N (%)** | **Patients without CD**  **(N=321)** | **Patients with CD***  **(N=7)** | **P-value§** |
| --- | --- | --- | --- |
| Only NSAID and intra-articular CS | 78 (24.3) | 3 (42.9) | 0.37 |
| cDMARDs | 233 (72.6) | 4 (57.1) | 0.4 |
| bDMARDs | 127 (39.6) | 2 (28.6) | 0.7 |

JIA: juvenile idiopathic arthritis; CD: celiac disease; NSAID: nonsteroidal anti-inflammatory drugs; CS: corticosteroid; cDMARDs: conventional disease-modifying anti-rheumatic drugs; bDMARDs: biological disease-modifying anti-rheumatic drugs.

*Pharmacological treatment in patients with JIA and CD include only therapies administered before or at the time of CD diagnosis.

§ Fisher’s exact test
